# The trial to reduce antimicrobial use in nursing home residents with Alzheimer’s disease and other dementias: study protocol for a cluster randomized controlled trial

**DOI:** 10.1186/s13063-019-3675-y

**Published:** 2019-10-15

**Authors:** Andrea J. Loizeau, Erika M. C. D’Agata, Michele L. Shaffer, Laura C. Hanson, Ruth A. Anderson, Timothy Tsai, Daniel A. Habtemariam, Elaine H. Bergman, Ruth P. Carroll, Simon M. Cohen, Erin M. E. Scott, Erin Stevens, Jeremy D. Whyman, Elizabeth H. Bennert, Susan L. Mitchell

**Affiliations:** 1 Hebrew SeniorLife Hinda and Arthur Marcus Institute for Aging Research, 1200 Centre Street, Boston, MA 02131 USA; 2 Division of Infectious Diseases, Warren Alpert Medical School, Brown University, Providence, RI USA; 3 Department of Statistics, University of Washington, Seattle, WA USA; 4 Division of Geriatric Medicine, Palliative Care Program, Chapel Hill, NC USA; 5 School of Nursing, University of North Carolina, Chapel Hill, NC USA; 6 Division of Palliative Care and Geriatrics, Department of Medicine, Massachusetts General Hospital, Boston, MA USA; 7 Department of Medicine, Beth Israel Deaconess Medical Center, Harvard Medical School, Boston, MA USA; 8 Postgraduate Medical Education, Harvard Medical School, Boston, MA USA

**Keywords:** Dementia, Palliative care, Infections, Antimicrobials, Decision-making, Nursing homes, Implementation, Cluster randomized trial, Pragmatic trial, Goals of care

## Abstract

**Background:**

Infections are common in nursing home (NH) residents with advanced dementia but are often managed inappropriately. Antimicrobials are extensively prescribed, but frequently with insufficient evidence to support a bacterial infection, promoting the emergence of multidrug-resistant organisms. Moreover, the benefits of antimicrobials remain unclear in these seriously ill residents for whom comfort is often the goal of care. Prior NH infection management interventions evaluated in randomized clinical trials (RCTs) did not consider patient preferences and lack evidence to support their effectiveness in ‘real-world’ practice.

**Methods:**

This report presents the rationale and methodology of TRAIN-AD (Trial to reduce antimicrobial use in nursing home residents with Alzheimer’s disease and other dementias), a parallel group, cluster RCT evaluating a multicomponent intervention to improve infection management for suspected urinary tract infections (UTIs) and lower respiratory tract infections (LRIs) among NH residents with advanced dementia. TRAIN-AD is being conducted in 28 facilities in the Boston, USA, area randomized in waves using minimization to achieve a balance on key characteristics (*N* = 14 facilities/arm). The involvement of the facilities includes a 3-month start-up period and a 24-month implementation/data collection phase. Residents are enrolled during the first 12 months of the 24-month implementation period and followed for up to 12 months. Individual consent is waived, thus almost all eligible residents are enrolled (target sample size, *N* = 410). The intervention integrates infectious disease and palliative care principles and includes provider training delivered through multiple modalities (in-person seminar, online course, management algorithms, and prescribing feedback) and an information booklet for families. Control facilities employ usual care. The primary outcome, abstracted from the residents’ charts, is the number of antimicrobial courses prescribed for UTIs and LRIs per person-year alive.

**Discussion:**

TRAIN-AD is the first cluster RCT testing a multicomponent intervention to improve infection management in NH residents with advanced dementia. Its findings will provide an evidence base to support the benefit of a program addressing the critical clinical and public health problem of antimicrobial misuse in these seriously ill residents. Moreover, its hybrid efficacy-effectiveness design will inform the future conduct of cluster RCTs evaluating nonpharmacological interventions in the complex NH setting in a way that is both internally valid and adaptable to the ‘real-world’.

**Trial registration:**

ClinicalTrials.gov, NCT03244917. Registered on 10 August 2017.

**Electronic supplementary material:**

The online version of this article (10.1186/s13063-019-3675-y) contains supplementary material, which is available to authorized users.

## Background

More than 5 million Americans are afflicted by Alzheimer’s disease and related dementias [1]. Suspected infection episodes are hallmarks of the end-stage of this disease [2–6]. Observational research from the nursing home (NH) setting, where advanced dementia patients commonly reside, reveal that these episodes are widely mismanaged, leading to adverse patient and public health outcomes [4–10].

Antimicrobials are extensively prescribed to NH residents with advanced dementia [3–5, 7, 11], most often in the absence of clinical evidence to support a bacterial infection [5, 7, 8, 12, 13]. Antimicrobial exposure is the main risk factor for multidrug-resistant organisms (MDROs) [14, 15]. NH residents with advanced dementia are three times more likely to be colonized with MDROs compared with other residents [16]. Moreover, as these residents have advanced dementia, evidence suggests they may not clinically benefit from antimicrobials [4, 17–19]. Comfort is the goal of care for most of these patients [5, 6, 20], and the risks and burdens associated with work-up and treatment of suspected infections generally do not promote that goal, particularly when hospitalization is involved [5, 18, 19].

Prior randomized controlled trials (RCTs) have demonstrated the efficacy of interventions designed to promote appropriate antimicrobial prescribing in the NH setting [21–28], but did not focus on advanced dementia patients or integrate goals of care. Moreover, even when proven to be efficacious, these previously tested interventions have not been adopted into practice. This lack of uptake may stem, in part, from the fact that these RCTs were primarily explanatory (versus pragmatic) in design, such that implementation was done by the research team with little integration into the NH culture.

Pragmatic RCTs are designed to test the effectiveness of interventions as they would be implemented in ‘real-world’ practice, and are well-suited to evaluate multicomponent behavioral interventions in the NH. Many RCTs are not strictly explanatory or pragmatic but include features of both designs. The Pragmatic-Explanatory Continuum Indicator Summary-2 (PRECIS-2) framework helps evaluate where key trial design features fall on the continuum between the two approaches [29]. Similarly, the National Institutes of Health (NIH) stage model describes six stages of behavioral intervention development (0–V), whereby stage III is characterized as a hybrid between an efficacy (i.e., more explanatory) and effectiveness (i.e., more pragmatic) trial [30].

The Trial to reduce antimicrobial use in nursing home residents with Alzheimer’s disease and other dementias (TRAIN-AD) is an ongoing NIH-funded cluster RCT that began in 2017. TRAIN-AD is evaluating a multicomponent intervention to improve the management of suspected infections in NH residents with advanced dementia. The intervention uniquely merges best practices in infectious diseases and palliative care. By applying both explanatory and pragmatic features, the design aims to evaluate the ‘real-world efficacy’ of a complex intervention in the NH setting. The objectives of this report are to describe the rationale and methodology of TRAIN-AD and evaluate its design features on the explanatory–pragmatic continuum using PRECIS-2.

## Methods

### Design

TRAIN-AD is a parallel group, cluster RCT testing a multicomponent intervention to improve infection management for suspected urinary tract infections (UTIs) and lower respiratory tract infections (LRIs) among NH residents with advanced dementia in 28 facilities in the Boston, USA, area (*N* = 14 facilities/arm). Its design complies with the Standard Protocol Items: Recommendations for Interventional Trials (SPIRIT) guidelines for clinical trial protocols and the Consolidated Standards of Reporting Trials (CONSORT) extension guidelines for cluster trials (see Additional file 1 and Additional file 2). The trial began in August 2017 and will be completed in June 2021. Its conduct was approved by the Institutional Review Board (IRB) at Hebrew SeniorLife.

### Facilities recruitment, randomization, and participation

**Fig. 1:**
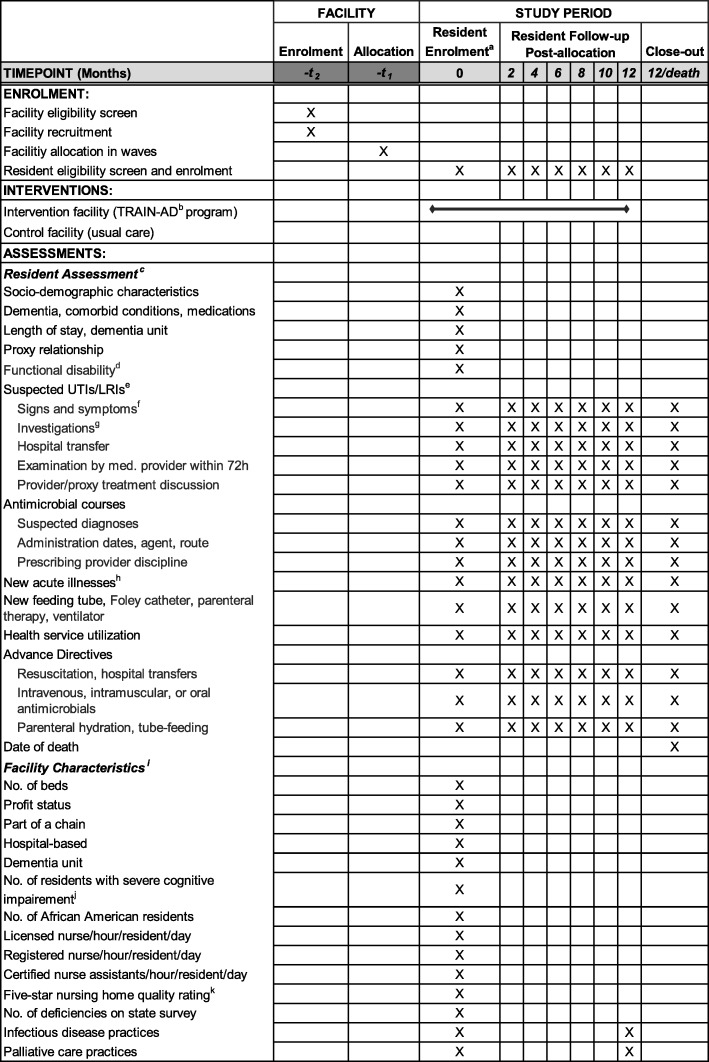
Schedule of enrolment, intervention, and assessments. ^a^The Institutional Review Board waived individual consent and thus almost all eligible residents are enrolled. ^b^The Trial to Reduce Antimicrobial Use in Nursing Home Residents with Alzheimer’s Disease and other Dementias. ^c^Resident assessments include chart reviews done at baseline (2-month look back), every 2 months and death, and each resident is followed for up to 12 months. ^d^Bedford Alzheimer Nursing Severity-Subscale (BANS-S: range 7–28, higher scores indicate more functional disability) [32]. ^e^Urinary tract (UTIs) and lower respiratory tract (LRIs) infections. ^f^Vital signs (e.g., temperature, respiratory rate), localized signs and symptoms (e.g., cough, hematuria) are recorded 24 h before or 72 h after initial documentation of the suspected infection. ^g^Investigations include urinalysis and urine, blood cultures, complete blood counts, and chest x-rays. ^h^Any new major acute illnesses other than infections such as bone fracture, stroke, or seizures. ^i^Facility data are collected from three sources: LTCfocus.org website [31]; Medicare.gov website (Medicare NH Compare) [33]; and survey administered to a senior administrator. ^j^Minimum Data Set Cognitive Function Scale (MDS CFS; score = 4 indicates severe impairment) [34]. ^k^Nursing Home Compare five-star rating (range 0–5; higher scores indicate better care quality)

**Fig. 2:**
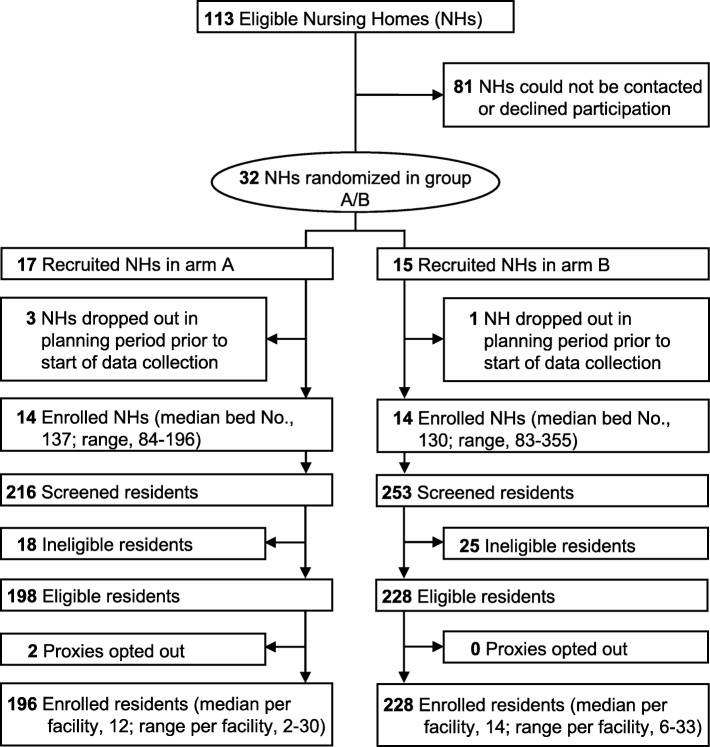
CONSORT diagram of facilities recruitment, randomization, and resident participation as of 25 July 2019. Anticipated completion of resident enrollment is 11 June 2020. Group A and B represent each trial arm, whose identity remains masked

Eligible NHs have greater than 60 beds and are located within 60 miles of Boston [31]. Study information is mailed to senior administrators, who are telephoned 1 week later by a research team member to solicit their facility’s participation, including their agreement to randomize their facility to either the intervention or control arm (Figs. 1 and 2).

**Fig. 3:**
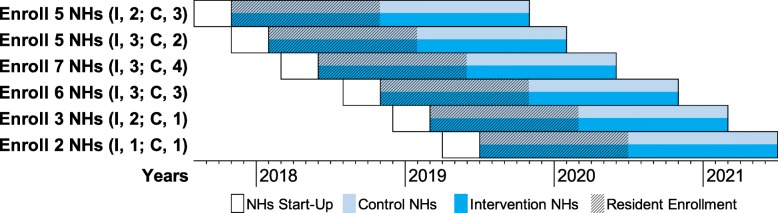
Study timetable of waves of intervention (I) and control (C) nursing home (NH) recruitment

The NH is the randomization unit since the intervention is delivered at the facility level as it would be in the ‘real world’. Facilities are recruited and randomized in waves. Originally, each wave was to be staggered by 4 months with six NHs recruited and randomized per wave (three NHs per arm). This scheme has been modified slightly to accommodate facility drop-outs and achieve resident recruitment targets (Fig. 3). Once a group of eligible NHs is recruited for a given wave, their identification numbers are sent to a statistician who uses a computer-generated algorithm to randomly allocate NHs either to the control or intervention arm, masked as A/B. Random allocation is conducted using minimization, an approach which minimizes imbalances of selected NH characteristics between study arms [35, 36].

The following NH characteristics ascertained from the Long-Term Care (LTC) focus database [31] were chosen for minimization based on the literature [5, 6, 37] as potentially associated with advanced dementia care: for-profit status; number of residents with severe cognitive impairment based on the Cognitive Function Scale (CFS) [34]; and number of African American residents. Facilities are categorized as having higher representation of severely cognitively impaired and African American residents based on median values of all eligible NHs.

For the first wave of six NHs, all possible ways of assigning three NHs to each arm were considered and the assignment that minimized imbalances across the three potential confounders was selected. For subsequent waves, all potential assignments of the new NHs and balance scores accumulating from previous waves were jointly considered. If multiple assignments yielded equivalently best-balance scores, an assignment was chosen randomly. Four randomized NHs dropped out prior to the initiation of data collection and were replaced in subsequent waves of randomization. Balance scores were recalculated to account for the removal of these facilities.

Once randomized, facilities are involved for 27 months, including a 3-month start-up period and a 24-month intervention implementation/data collection phase. At the intervention facilities, a senior administrator designates an NH staff member (e.g., the infection preventionist or the director of nursing) as the TRAIN-AD site champion to work with the research team to lead the intervention implementation and serve as the on-site TRAIN-AD resource for providers.

### Participants

Enrollment of NH residents began in October 2017 and will be completed in June 2020. In all facilities, residents are enrolled for the first 12 months of the 24-month intervention implementation/data collection phase, ensuring the opportunity for 12 months of follow-up for every resident (Figs. 1 and 3).

Resident eligibility criteria include: 1) age ≥ 60 years; 2) dementia (any type); 3) Global Deterioration Scale (GDS) score of 7 (range 1–7, higher scores indicate worse dementia) [38]; 4) length of NH stay > 90 days; and 5) had a designated English-speaking proxy (i.e., a relative or legal guardian designated as responsible for making healthcare decisions on behalf of the resident). Residents with a GDS score of 7 have profound memory deficits, speak < 5 words, are functionally dependent, incontinent, and nonambulatory. During the facility start-up period and every 2 months up to 12 months, research assistants (RAs) interview nurses on each unit to identify residents with dementia and a GDS score of 7, and meeting the criteria for age, dementia diagnosis, length of stay, and confirmed proxy availability by chart review.

The IRB waived formal individual consent as the trial was deemed to be of minimal risk, the protocol involves no direct contact between the research team and residents, all data are ascertained from resident medical charts, and the program is delivered by providers at the facility level. Designated proxies were still provided with essential information about the study and the opportunity to refuse data collection from the resident’s chart. Flyers posted throughout all facilities provide study information and who to contact if proxies do not want data being collected from their residents’ charts (i.e., ‘opt-out’). Proxies of eligible residents in the intervention arm are also mailed this flyer. In the absence of an opt-out request, all eligible residents are enrolled and followed throughout the data collection period. To date, only two proxies of 426 eligible residents have opted out (0.5%) (Fig. 2).

### Providers in the intervention arm

At each intervention facility, the TRAIN-AD champion identifies ‘targeted’ providers who have primary care responsibilities for advanced dementia residents defined as follows: 1) nurses (registered or licensed practical nurses) who work a minimum of two shifts most weeks caring for advanced dementia residents; and 2) prescribing medical providers (physicians, nurse practitioners and physician assistants) who have a minimum of two advanced dementia residents on their regular patient panel.

### Intervention components

The TRAIN-AD intervention has multiple components (Table 1) designed with redundancies to maximize learning opportunities and content reinforcement, and includes: 1) algorithms displayed on posters and laminated pocket cards to guide clinical management of suspected UTIs and LRIs in advanced dementia; 2) an online interactive course for providers entitled ‘Management of Infections in Advanced Dementia’; 3) in-person provider training seminar; 4) laminated pocket cards offering quick tips for providers to communicate with proxies; 5) prescribing feedback reports for medical providers; and 6) a booklet entitled ‘Infections in Advanced Dementia: What the Family Should Know about Treatment Decisions’ for proxies. Each component integrates best practices in infectious diseases and palliative care. All components were developed through an iterative process with experts in geriatrics, infectious diseases, and palliative medicine, guided by the literature [4–6, 11, 12, 16–19, 39, 40], and refined based on peer review and pilot testing.

**Table 1:** TRAIN-AD intervention components integrating infectious disease and palliative care principles

Provider training	Format	Learners
Algorithms	Poster and pocket card displaying algorithms to guide clinical management of suspected UTIs and LRIs in advanced dementia that consider patient preferences	Targeted providers^a^
Online course	Online, 1-h interactive course hosted by the HMS DCE presenting management principles using virtual patient cases, algorithms, and communication demonstration videos	Targeted providers
Seminar^b^	In-person, 1-h training seminar led by a physician, dually boarded in geriatrics and palliative care, presenting management principles and program components	Targeted providers
Communication tips	Pocket card, based on VitalTalk [39] framework, displaying quick tips to communicate with proxies about managing infections in advanced dementia and goals of care	Targeted providers
Prescribing feedback reports	Every 2 months, reports prepared by the research team for medical providers on the appropriateness of their antimicrobial initiation for suspected UTIs and LRIs	Medical providers, site champion
Proxy education booklet	Booklet for proxies of nursing home residents providing information on infections management and preference-based decision-making in advanced dementia	Proxies

^a^Targeted providers are: 1) nurses (registered or licensed practical nurses) who work a minimum of two shifts most weeks caring for advanced dementia residents; and 2) prescribing medical providers (physicians, nurse practitioners and physician assistants) who have a minimum of two advanced dementia residents on their regular patient panel

^b^Providers unable to attend the seminar are offered a 10-min one-on-one mini-orientation

*HMS DCE* Harvard Medical School Department of Continuing Education, *LRI* lower respiratory tract infection, *TRAIN-AD* Trial to reduce antimicrobial use in nursing home residents with Alzheimer’s disease and other dementias, *UTI* urinary tract infection

**Fig. 4:**
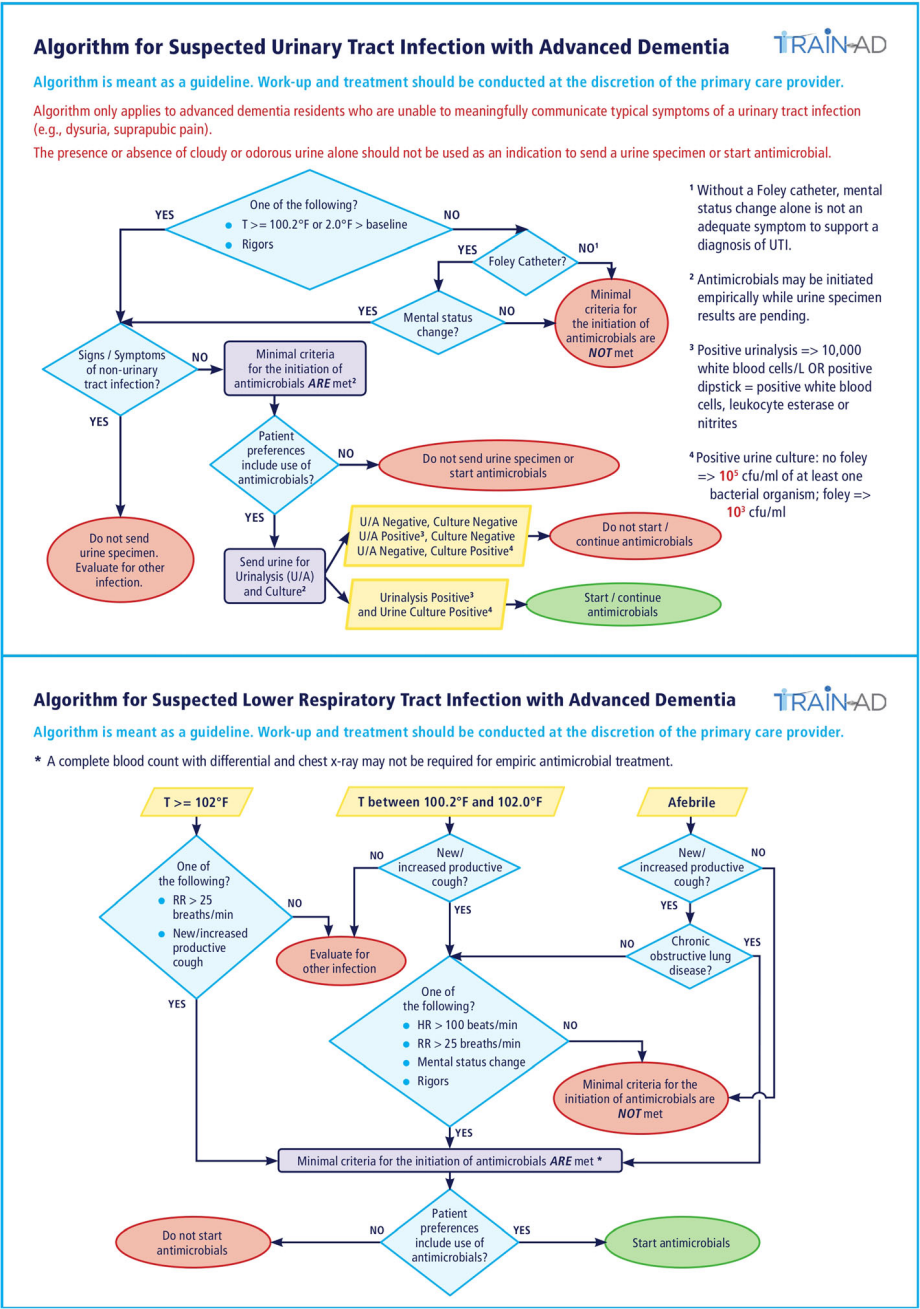
Algorithms for managing suspected UTIs and LRIs infections in nursing home residents with advanced dementia. CFU colony-forming units, HR heart rate, LRI lower respiratory tract infection, RR respiratory rate, T temperature, U/A urinalysis, UTI urinary tract infection

Two management algorithms were developed (Fig. 4), one for suspected UTIs and a second for suspected LRIs. In both, decisions to start antimicrobials are guided by two main considerations: 1) whether minimal criteria for antimicrobial initiation is met [40]; and 2) whether the acre preferences of patients include antimicrobial use. The consensus-based criteria for starting antimicrobials were based on published guidelines and slightly modified for advanced dementia residents who typically cannot communicate many symptoms of infections (e.g., dysuria) [40, 41]. Posters displaying the algorithms are hung in nursing units in intervention facilities and laminated 4 × 5 in. pocket cards are given to targeted providers. These materials clearly state that management decisions are ultimately up to the discretion of the medical providers.

‘Infection Management in Advanced Dementia’ is a peer-reviewed, online, 1-h course co-designed by experts in advanced dementia (SLM) and infectious diseases (EMCD) and hosted by the Harvard Medical School Department of Continuing Education [42]. The course consists of four virtual patient cases that illustrate principles on managing suspected UTIs (two cases) and LRIs (two cases) in advanced dementia, including the use of the TRAIN-AD algorithms. To demonstrate best practice communication strategies, the course includes three short videos in which paid actors conduct scripted conversations for challenging scenarios between medical providers and proxies, including: 1) a proxy insisting the resident gets antimicrobials when there is no clinical indication; 2) a proxy considering options for management of a suspected LRI; and 3) advanced care planning for infection management. The video dialogues were informed by the VitalTalk communication program [39], where a 10-item pre- and post-test ascertains the learner’s knowledge, with a minimum post-test score of 75% required for course completion.

The in-person, 1-h seminar conducted at the NHs is aimed at the targeted providers. One of three physician educators (EMES, ES, JDW), boarded in both geriatrics and palliative medicine, lead the seminars at each facility using the same content and structure. A didactic slide presentation explains the program’s rationale, components, management principles, and communication tips. The didactic portion is followed by open discussion and step-by-step instructions about how to access the online course. Laminated pocket cards for providers with quick communication tips based on the VitalTalk Communication framework [39] are also distributed.

Provider feedback reports are created by the research team and distributed to prescribing medical providers and TRAIN-AD champions every 2 months by email. The individualized report was designed to motivate behavior change to reduce inappropriate prescribing. The report presents cases in the prior 2 months for which the provider prescribed antimicrobials for suspected UTIs or LRIs to advanced dementia residents when minimal criteria for treatment initiation were absent. This determination was made by the research team based on documented signs and symptoms abstracted by research assistants from the residents’ charts [40].

Finally, proxies in the intervention NHs are sent ‘Infections in Advanced Dementia: What the Family Should Know about Treatment Decisions’, a six-page booklet with information pertaining to suspected infections in advanced dementia, treatment options for these episodes and their risks and benefits, and guidance about how to align treatment decisions with goals of care. The booklet, written at grade 6 reading level, was edited by actual family members of individuals with advanced dementia.

### Intervention implementation

A structured 3-month start-up period precedes the 24-month implementation period at all intervention facilities. Each month during the start-up period, the research implementation team (i.e., research nurse, project director, and RA) meet in-person with the NH leadership who typically includes the TRAIN-AD champion, director of nursing (if not the champion), senior administrator, and other staff members, such as clinical education specialists. The start-up goals are to establish a relationship between the research and NH teams, review all program components, cooperatively plan its implementation, and adapt the program to each NH’s culture and workflow. Between monthly meetings, the research nurse and site champion meet by telephone weekly to ensure planning is on track. A TRAIN-AD site manual describing all program components is provided to senior leadership. In addition, a TRAIN-AD resource binder is given to the champion which includes key scientific articles about infection management in advanced dementia, links to relevant authoritative websites, and documents from the VitalTalk communication program [39]. The resource binder is meant to provide the champion with readily available information to support their efforts to promote informed decision-making by providers and proxies.

In the start-up period, the champion generates a list of the targeted providers and their email addresses. In the final month of the start-up, these providers are mailed a TRAIN-AD orientation package. Providers can refuse to participate in any or all of the intervention components by using the contact information provided in the orientation package. Targeted providers are invited to attend the in-person training seminar which is held in the final 2 weeks of the start-up period and offered twice on the same day to reach various shifts. Providers unable to attend the seminar are offered a 10-min one-on-one mini-orientation delivered by either the champion or a research team member.

After receiving instructions about accessing the online course, each targeted provider is sent an email with a hyperlink to the course. Providers are asked to complete the course within 4 weeks but are ultimately given extensions for up to 3 months. Providers completing the course receive a $50 American Express gift card and 1 Continuing Medical Education for medical providers or 1 Continuing Education Unit for nurses. An RA tracks which providers complete the course in real time. Each week for up to 3 months, the RA emails reminders to noncompliant providers and sends their names to the champion to encourage participation. To further incentivize participation, a Chromebook is raffled among providers in facilities achieving 67% course completion.

Following the training seminar, which marks the official start to the intervention, management algorithms posters are hung in the nursing units caring for advanced dementia residents (approximately two posters/facility). Family booklets are mailed to proxies at the time of resident enrollment. While the research team manage the practical aspects and costs of this mailing, the letter is printed on the NH’s stationary and signed by a senior NH administrator. Champion and providers are encouraged to review the booklets with proxies at care planning meetings or the next opportunity. The provider prescribing feedback reports are emailed every 2 months during the 24-month implementation period directly to each prescribing provider and to their respective champion.

Every 6 months during the implementation period, newly hired targeted providers are identified by the champions and undergo the aforementioned on-boarding process. The research team offers on-site orientation seminars every 6 months for new providers to which ongoing providers are also invited to solicit their feedback and problem solve ongoing issues. Additionally, every 6 months, in-person meetings are held between the research team and NH leadership to review all program components and optimize implementation.

**Fig. 5:**
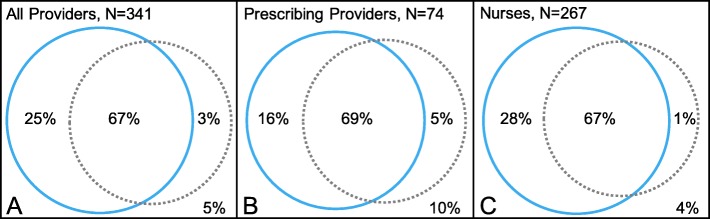
Proportions of targeted providers completing the training seminar (blue circle) and online course (dotted circle). The Venn diagrams display proportions for all providers (**a**; *N* = 341), prescribing providers (physicians, nurse practitioners, and physician assistants) (**b**; *N* = 74), and nurses (**c**; *N* = 267). The numbers correspond to the 13 facilities which have completed the 3-month start-up period as of 31 July 2019 and exclude those from the remaining facility in which providers are currently offered the training seminar and online course

Measures of implementation protocol adherence are generated in real time. Venn diagrams are used to display the proportions of targeted providers who attended a training seminar (full or mini) and/or completed the online course. For example, as shown in Fig. 5, to date, among providers who have been in the trial for at least 3 months, adherence results for completing either the seminar or course are: all providers, *N* = 323/341 (95%); prescribing providers, *N* = 67/74 (90%); nurses, *N* = 256/267 (96%). Prescribing feedback reports provide another ongoing measure of protocol adherence and opportunity for reinforcement. The NH leadership is encouraged to integrate the TRAIN-AD compliance measures into their regular Quality Assurance and Performance Improvement activities [43].

### Control arm

After randomization, control facilities are informed of their group assignment. Control facilities employ usual care for suspected infections. During the start-up period and whenever needed over the 24-month implementation period, in-house services are held to orient NH staff to resident screening and data collection procedures. No restrictions are placed on either the control or intervention facilities with regard to their use of other antimicrobial stewardship, advance care planning, or palliative care programs.

### Masking

Research implementation team members cannot be masked to the study arms. The two RAs who conduct resident chart abstractions, which include all outcome data, are masked and not told that data are being collected for an RCT. The principal investigator (SLM), statistician (MLS), and data programmers (DAH, TT) are masked.

### Data collection and elements

Resident data collection is the same in both arms unless otherwise stated. Data are collected at baseline, every 2 months up to 12 months, and within 30 days of death (Fig. 1). Baseline data abstracted from the residents’ charts include: socio-demographic characteristics (age, gender, race (white versus other), ethnicity, primary language, education, religion, marital status), dementia type (Alzheimer’s disease versus other), medical comorbidities, daily medications, length of NH stay, residing in a special care dementia unit, and proxy relationship to resident. At baseline, nurses quantify the residents’ functional status in a 1-min interview using the Bedford Alzheimer Nursing Severity-Subscale (BANS-S; range 7–28, higher scores indicate more functional disability) [32].

At all follow-up assessments, details about any suspected UTIs or LRIs during the prior 2 months are abstracted from the charts independently by two RAs. Inconsistencies are resolved by the project director. Identification of suspected UTIs or LRIs is based on specific documentation in provider progress notes or inferred from tests ordered (e.g., urine culture). For such episodes, the following data are abstracted: vital signs (e.g., temperature, respiratory rate), localized signs and symptoms (e.g., cough, hematuria) recorded 24 h before or 72 h after initial documentation of the suspected infection, investigations and results if available (e.g., urinalysis and urine, blood cultures, complete blood counts, chest x-rays), whether the resident was transferred to the hospital (hospitalization or emergency department visit), and whether a medical provider examined the resident within 72 h of presentation. Details about documented discussions between providers and proxies related to these episodes are also ascertained including the providers’ discipline (e.g., nurse, physician), and mode of discussion (in person or by phone).

Details about all antimicrobial courses prescribed for suspected UTIs or LRIs, as well as any other indications, are ascertained from the Medication Administration Records at all assessments including: suspected diagnoses, administration dates, agent, route (oral, intramuscular, intravenous, or per gastrostomy tube), and the prescribing provider including their discipline (physician, nurse practitioner or physician assistant). For each suspected UTI and LRI treated with antimicrobials, a determination is made by the research team as to whether the minimal criteria to initiate treatment were present based on documented signs and symptoms. This determination is guided by operationalizing expert-based consensus guidelines [40, 41] as was done in prior research [5, 41].

Additional data collected from the charts at all assessments include: occurrence of new major acute illnesses in the prior 2 months (e.g., bone fracture, stroke, or seizures); use of feeding tubes, Foley catheters, parenteral therapy, and ventilators; and health service utilization. Charts are abstracted for discussions between providers and proxies about preferences for management of suspected infections, and advance directives to withhold any of the following: resuscitation, hospital transfers, antimicrobials (intravenous, intramuscular, or oral), parenteral hydration, and tube feeding. In case of death, the date is ascertained.

Facility data are collected at baseline for descriptive purposes from three sources: the LTCfocus.org website [31]; the Medicare.gov website (Medicare NH Compare) [33]; and a survey administered to a senior administrator (Fig. 1). Facility variables from LTC focus include number of beds, for-profit status, part of a chain, hospital-based facility, dementia unit, number of residents with severe cognitive impairment [34], number of African American/white residents, and number of registered and licensed nurse and certified nurse assistant hours per resident per day. Variables obtained from Medicare NH Compare were five-star NH quality rating, which is a composite of rankings of health inspections, quality indicators, and staffing (total range 0–5, higher scores indicate better quality of care), and the number of health deficiencies on the previous state inspection [33]. The administrator survey is conducted by telephone or in person at baseline, 12 months, and 24 months to ascertain information about facility practices for infectious diseases and palliative care (see Additional file 3). Infectious disease questions include the presence of an infection preventionist, antibiotic stewardship program utilization, standardized protocols to manage UTIs and LRIs, and an on-site capability to manage infections (e.g., intravenous administration of antimicrobials, chest x-rays, on-site medical providers). Palliative care practice questions include standardized advance directive forms, access to hospice and/or palliative care consultations, and approach-to-proxy discussions about infection management.

### Outcomes

The primary trial outcome is the number of antimicrobial courses for suspected UTIs and LRIs per person-year over 12 months. The values are represented as per person-year to provide comparable comparisons between arms as follow-up time (resident exposure time expressed in years) may differ due to mortality. A 3-day treatment-free interval defines a separate antimicrobial course. Episodes in which more than one antimicrobial are given the same day are counted as a single course.

Secondary outcomes include the number of antimicrobial courses for suspected UTIs and LRIs when minimal criteria for initiation are absent per person-year over 12 months, and acquiring a new directive to withhold antimicrobials and the total number of potentially burdensome procedures (bladder catheterizations, blood draws, chest x-rays, and hospital transfers) used to evaluate a suspected UTI or LRI per person-year.

### Sample size calculation

The sample size was calculated as 410 residents from 28 facilities (205 residents from 14 facilities/arm) to provide at least 90% power to detect an absolute reduction in antimicrobial courses per person-year of 0.38 in intervention (versus control) arms. An ordinary Poisson regression sample size calculation was multiplied by the cluster design effect to calculate the number of residents required per group [44]. It assumed two-sided testing, 5% type I error rate, intraclass correlation coefficient for facilities of 0.01, 15 residents/facility, 0.91 courses/resident which will not meet minimal criteria for treatment in the control arm, and an average of 0.79 person-years contributed by each resident based upon prior research [5].

### Statistical analysis

Analyses will be performed using SAS 9.4 (SAS Institute, Inc., Cary, NC, USA), STATA 13.1 (College Station, TX, USA), and R 3.4.1 (R Foundation for Statistical Computing, Vienna, Austria). Descriptive analyses will include frequencies for categorical variables and means/medians with standard deviations (SDs)/interquartile ranges for continuous variables. Outcome analyses will be conducted at the resident level and follow intention-to-treat principles. Adjustment for baseline imbalances in resident features will be considered as sensitivity analysis, as needed. While no effect on mortality is anticipated, analyses will account for the competing risk of death and differential follow-up time due to mortality. Robust estimates of variance will account for residents clustered within NHs with bootstrap-based adjustment since the number of clusters is fewer than 30 [45]. A negative binomial-logit regression model will be used to compare the primary outcome (number of antimicrobial courses for suspected UTIs and LRIs per person-year over 12 months) between trial arms. A potential excess of residents not receiving antimicrobials will be accommodated by using a two-stage process—a logit model examining the probability of receiving at least one antimicrobial course and, among those, a truncated negative binomial model examining the number of courses. Length of follow-up (time to death, dropout, or study completion) will be included as an offset. The model’s logit portion will generate odds ratios and its negative binomial portion will generate rate ratios, both with 95% confidence intervals (CIs). A two-sided likelihood ratio test will jointly evaluate whether the intervention reduced the probability of having at least one antimicrobial course and, given any antimicrobial use, the number of courses. The same analytic approach will be used to compare two of the secondary outcomes between study arms: 1) number of antimicrobial courses used for suspected UTIs and LRIs when minimal criteria for initiation were absent per person-year over 12 months; and 2) total number of burdensome procedures used to evaluate a suspected UTI or LRI per person-year whereby burdensome procedures will include bladder catheterizations, blood draws, chest x-rays, and hospital transfers. Exploratory analyses will examine each intervention separately.

Cox proportional hazards regression will be used to compare the acquisition of advance directives to restrict antimicrobial use between study arms. The outcome will be defined as the time between the date of the baseline assessment of the resident to the date at which a more restrictive directive was documented. Acquisition from a less to a more restrictive advance directive for antimicrobial use will be defined as including any of the following: 1) having no advance directives to withhold antimicrobials to acquiring a directive to withhold antimicrobials by any modality (oral, intramuscular, intravenous); 2) only having a directive to withhold intravenous antimicrobials to acquiring a directive to withhold intramuscular or oral antimicrobials; and 3) having directives to only withhold intravenous and/or intramuscular antimicrobials to acquiring a directive to withhold oral antimicrobials. Residents with directives restricting all routes of antimicrobial use at baseline will be excluded from these analyses. Residents who died, dropped-out, or completed the study without acquisition of more restrictive directives will be censored. A sensitivity analysis will be conducted to examine if the potential competing risk of death impacted the estimates of cumulative incidence using the Fine-Gray regression model [46]. Hazard ratios and 95% CIs will be generated from these analyses.

#### Design features on the explanatory–pragmatic continuum

The TRAIN-AD design features were assessed along the nine domains of the explanatory–pragmatic continuum of the PRECIS-2 framework [29]. The domains (eligibility, recruitment, setting, organization, flexibility–delivery, flexibility–adherence, follow-up, primary outcome, and primary analysis) are scored as very explanatory (1), rather explanatory (2), equally explanatory and pragmatic (3), rather pragmatic (4), and very pragmatic (5). The TRAIN-AD rating was determined by the consensus of two TRAIN-AD investigators (AJL, SLM).

The assessment of the TRAIN-AD design features along the explanatory–pragmatic continuum in the nine domains of the PRECIS-2 framework revealed the following scores: eligibility (4), recruitment (3), setting (3), organization (3), flexibility–delivery (3), flexibility–adherence (3), follow-up (3), primary outcome (4), and primary analysis (5). The rationales for these scores are presented in Table 2.

**Table 2:** Assessment of the TRAIN-AD design features along the explanatory–pragmatic continuum in the nine domains of the PRECIS-2 framework

Domains (score)^a^	Rationales
Eligibility (4)	Explanatory: The trial is limited to residents in facilities in the Boston area Pragmatic: All eligible residents are enrolled and are typical of nursing home (NH) residents with advanced dementia
Recruitment (3)	Explanatory: Considerable effort is required by the research team to recruit NHs and identify eligible residents Pragmatic: Once identified, all eligible residents are enrolled
Setting (3)	Explanatory: Participant NHs are limited to those that agreed to participate and the Boston area. NHs that refuse to participate and those in other regions may differ from participating facilities (demographics, culture, approach to care) Pragmatic: Participant NHs are typical of those caring for advanced dementia residents
Organization (3)	Explanatory: The research team designed the intervention structure, provides resources, delivers the main training seminar, and offers incentives to complete the online course Pragmatic: The intervention implementation is done in partnership with the site champion. The intervention is not resource-intensive and has high potential for implementation outside of a research trial, as it aligns closely with federal mandates for antimicrobial stewardship programs [47]. Moreover, the online course is publicly available and thus not limited to TRAIN-AD providers
Flexibility–delivery (3)	Explanatory: The intervention delivery is largely dictated by the research protocol, such as the timing of training seminars, time frame to complete online course, mailing of booklets to families, and provision of feedback reports Pragmatic: Aspects of the implementation delivery are adaptable; e.g., providers can do the training seminar in an abbreviated one-on-one orientation rather than attend the group session, and other antimicrobial stewardship and/or advance care planning programs outside of the TRAIN-AD protocol are permitted to continue
Flexibility–adherence (3)	Explanatory: Provider participation is voluntary but closely monitored and reinforced as part of the research protocol using compensations and provider feedback reports Pragmatic: Treatment decisions are left to the discretion of clinical providers and not mandated by the research protocol
Follow-up (3)	Explanatory: All participant follow-ups are rigorously conducted by two research assistants Pragmatic: Follow-ups only involve abstraction of information from the residents’ charts collected as part of clinical care
Primary outcome (4)	Pragmatic: The primary outcome closely aligns with key measures of federal mandated antimicrobial stewardship programs [47], and is thus highly relevant to key stakeholders and can be ascertained using electronic medical charts
Primary analysis (5)	Pragmatic: it will follow the intention-to-treat principle using all available data. Poorly adherent facilities will not be excluded from the analysis

^a^Each domain is scored as follows: very explanatory (1), rather explanatory (2), equally explanatory and pragmatic (3), rather pragmatic (4), and very pragmatic (5)

*PRECIS-2* Pragmatic–Explanatory Continuum Indicator Summary 2 [29], *TRAIN-AD* Trial to Reduce Antimicrobial use In Nursing home residents with Alzheimer’s disease and other Dementias

## Discussion

TRAIN-AD is a unique, multisite, cluster RCT testing a multicomponent intervention to improve infection management in NH residents with advanced dementia that merges best clinical practices in infectious diseases and palliative care. Its design includes both explanatory and pragmatic features and can best be described as a hybrid efficacy–effectiveness trial [30]. The trial’s findings will provide the evidence base to support the benefit of the TRAIN-AD program to address the critical clinical and public health problem of antimicrobial misuse in these seriously ill residents. Moreover, its methodology will inform future conduct of cluster RCTs evaluating multicomponent nonpharmacological interventions in the complex NH setting such that the findings are both internally valid and adaptable to the ‘real-world’.

The rationale underlying TRAIN-AD builds on the well-recognized need to improve infection management in the NH setting [13, 48], as reflected in the recent request from the Centers for Disease Control (CDC) to expand antimicrobial stewardship activities in NHs [47]. However, TRAIN-AD is the first such initiative targeting the specific circumstances of residents with advanced dementia for whom the need to improve infection management is compelling. A growing body of literature has also highlighted concerns about infection management at the end of life [49]. Although many NH residents have advanced illnesses, the CDC initiative and most standard NH antimicrobial stewardship programs focus solely on infectious disease guidelines [21–28, 47]. The TRAIN-AD program goes further by integrating palliative care principles into its management algorithms, training materials, and evaluation metrics. Moreover, the TRAIN-AD program includes state-of-the art communication training [39], addressing the need of NH providers for guidance on discussing infection management decisions with families.

The structure and implementation strategy of the TRAIN-AD intervention builds on the experience of prior RCTs of NH antimicrobial stewardship programs [21–28]. For example, most prior interventions with proven efficacy had multiple components to maximize learning opportunities and included prescribing feedback to providers to motivate behavior change [27, 28, 47, 48, 50, 51]. The TRAIN-AD program includes these features, but also attempts to address a major shortcoming of earlier interventions; namely, that even those with demonstrated efficacy have not been fully adopted into practice. To help ensure that TRAIN-AD is adoptable and sustainable, senior administration support is ascertained from the onset, and key stakeholders (e.g., medical providers, nurses, families) are engaged in learning, communication, monitoring and adoption throughout the 3-month start-up and 24-month implementation periods [27, 28, 52, 53]. Partnering with and supporting the champions to take ownership of parts of the implementation (e.g., provider training, review of feedback reports) is essential to this effort.

As revealed by our PRECIS-2 evaluation, TRAIN-AD has both pragmatic and explanatory features. As such, its design aligns best with stage III on the NIH stage model, characterized as the ‘real-life efficacy’ or ‘hybrid efficacy–effectiveness’ phase of intervention development [30]. Its more explanatory features prioritize the need to understand how the program impacts antimicrobial use when there is a high level of protocol adherence and rigorous outcomes ascertainment. The more pragmatic features support the likelihood that, if efficacious, the program could be integrated into actual practice within a larger NH system. Evaluation at this next stage of intervention development, such as a fully pragmatic trial or stage IV on the NIH stage model, will require some design adaptations such as linkage to electronic pharmacy data to ascertain antimicrobial use and perhaps tailoring of the intervention as guided by the TRAIN-AD implementation experience.

Two design features of TRAIN-AD are particularly instructive to the conduct of future cluster RCTs in NHs. First, with our staggered waves of NH enrollment, minimization proved to be a very practical approach to achieving balanced randomization of NH clusters based on key characteristics. Imbalances of potentially confounding characteristics of randomized units is a threat to the validity of cluster RCTs. Other approaches to attain balance, such as matching or stratification, are more problematic to employ with relatively few clusters, several potentially confounding variables, and a rolling approach to recruitment [36]. The second design feature worth highlighting is the approach to individual consents. The IRB permitted the waiver of informed consent based on federal regulations [54], an approach increasingly used in pragmatic trails [55]. Such a waiver increases recruitment and avoids unnecessary administrative procedures for participants and the research team. This pragmatic approach resulted in 99.5% of eligible residents being enrolled in TRAIN-AD to date, and is less onerous for the research team and will greatly enhance the generalizability of the findings.

Several limitations of the TRAIN-AD design merit comment. First, generalizability will be limited to the participating facilities in the Boston area. Second, clinical management of suspected UTIs and LRIs is ultimately up to providers who may or may not have incorporated TRAIN-AD principles. However, this approach reflects how clinical decision-making occurs in the ‘real-world’. Third, concurrent NH initiatives for infection management or palliative care may have occurred in parallel with the TRAIN-AD program; however, these activities are monitored and may be similar between trial arms.

As the first cluster RCT to evaluate an intervention to improve infection management in vulnerable NH residents with advance dementia, TRAIN-AD will advance the evidence base regarding the impact of such programs on antimicrobial use and end-of-life care, and also inform the methodology of conducting such trials of behavioral interventions in the NH setting. As a hybrid efficacy–effectiveness trial, the TRAIN-AD program will be well poised to advance to the next stage of investigation, namely a fully pragmatic trial embedded in an NH healthcare system.

## Trial status

This is protocol version 1.0, 31 July 2019. Enrollment of nursing home residents with advanced dementia began in October 2017 and will be completed in June 2020.

## Additional files

Additional file 1: The Trial to reduce antimicrobial use in nursing home residents with Alzheimer’s disease and other dementias (TRAIN-AD) using the Standard Protocol Items: Recommendations for Interventional Trials (SPIRIT) 2013 checklist. (PDF 127 kb)

Additional file 2: The Trial to reduce antimicrobial use in nursing home residents with Alzheimer’s disease and other dementias (TRAIN-AD) using the Consolidated Standards of Reporting Trials (CONSORT) checklist extension for cluster trials. (PDF 365 kb)

Additional file 3: The Trial to reduce antimicrobial use in nursing home residents with Alzheimer’s disease and other dementias (TRAIN-AD) administrator survey. (PDF 87 kb)

## Data Availability

The datasets generated and/or analyzed during the current study are not publicly available as the trial is not complete. Upon trial completion, data will be available from the Principal Investigator (SLM) on reasonable request.

## Abbreviations

BANS-S: Bedford Alzheimer Nursing Severity-Subscale
CDC: Centers for Disease Control
CFS: Cognitive Function Scale
CI: Confidence interval
CONSORT: Consolidated Standards of Reporting Trials
GDS: Global Deterioration Scale
IRB: Institutional Review Board
LRI: Lower respiratory tract infection
LTC: Long-Term Care
MDRO: Multidrug-resistant organisms
NH: Nursing home
NIH: National Institutes of Health
PRECIS-2: Pragmatic-Explanatory Continuum Indicator Summary-2
RA: Research assistant
RCT: Randomized controlled trial
SPIRIT: Standard Protocol Items: Recommendations for Interventional Trials
TRAIN-AD: Trial to reduce antimicrobial use in nursing home residents with Alzheimer’s disease and other dementias
UTI: Urinary tract infection
