# Supplementary material for: The trial to reduce antimicrobial use in nursing home residents with Alzheimer’s disease and other dementias: study protocol for a cluster randomized controlled trial
Source: Trials. 2019 Oct 15;20:594. doi: 10.1186/s13063-019-3675-y (PMC6794759; doi:10.1186/s13063-019-3675-y)
Supplement: Supplementary file 2 — The Trial to reduce antimicrobial use in nursing home residents with Alzheimer’s disease and other dementias (TRAIN-AD) using the Consolidated Standards of Reporting Trials (CONSORT) checklist extension for cluster trials. (PDF 365 kb) [file 13063_2019_3675_MOESM2_ESM.pdf]

Additional File 2. The Trial to Reduce Antimicrobial use In Nursing home residents with Alzheimer's disease and other Dementias (TRAIN-AD) using the CONsolidated Standards Of Reporting Trials (CONSORT) checklist extension for cluster trials

Table 1: CONSORT 2010 checklist of information to include when reporting a cluster randomised trial

Section/Topic	Item No	Standard Checklist item	Extension for cluster designs	Page No *
**Title and abstract**				
	1a	Identification as a randomised trial in the title	Identification as a cluster randomised trial in the title	**1**
	1b	Structured summary of trial design, methods, results, and conclusions (for specific guidance see CONSORT for abstracts)[1,2]	See table 2	**3, 4**
**Introduction**				
**Background and objectives**	2a	Scientific background and explanation of rationale	Rationale for using a cluster design	**5, 6**
	2b	Specific objectives or hypotheses	Whether objectives pertain to the the cluster level, the individual participant level or both	**6**
**Methods**				
**Trial design**	3a	Description of trial design (such as parallel, factorial) including allocation ratio	Definition of cluster and description of how the design features apply to the clusters	**6, 7**
	3b	Important changes to methods after trial commencement (such as eligibility criteria), with reasons		**NA**
**Participants**	4a	Eligibility criteria for participants	Eligibility criteria for clusters	**7-9**
	4b	Settings and locations where the data were collected		**6, 7; 15-18**
**Interventions**	5	The interventions for each group with sufficient details to allow replication, including how and when they were actually administered	Whether interventions pertain to the cluster level, the individual participant level or both	**10-15, Table 1**

**Outcomes**	6a	Completely defined pre-specified primary and secondary outcome measures, including how and when they were assessed	Whether outcome measures pertain to the cluster level, the individual participant level or both	**18**
	6b	Any changes to trial outcomes after the trial commenced, with reasons		**NA**
**Sample size**	7a	How sample size was determined	Method of calculation, number of clusters(s) (and whether equal or unequal cluster sizes are assumed), cluster size, a coefficient of intracluster correlation (ICC or *k*), and an indication of its uncertainty	**18, 19**
	7b	When applicable, explanation of any interim analyses and stopping guidelines		**NA**
**Randomisation:**				
**Sequence generation**	8a	Method used to generate the random allocation sequence		**7, 8**
	8b	Type of randomisation; details of any restriction (such as blocking and block size)	Details of stratification or matching if used	**7, 8**
**Allocation concealment mechanism**	9	Mechanism used to implement the random allocation sequence (such as sequentially numbered containers), describing any steps taken to conceal the sequence until interventions were assigned	Specification that allocation was based on clusters rather than individuals and whether allocation concealment (if any) was at the cluster level, the individual participant level or both	**7, 8, Figure 1**
**Implementation**	10	Who generated the random allocation sequence, who enrolled participants, and who assigned participants to interventions	Replace by 10a, 10b and 10c	**7**
	10a		Who generated the random allocation sequence, who	**7**

			enrolled clusters, and who assigned clusters to interventions	
	10b		Mechanism by which individual participants were included in clusters for the purposes of the trial (such as complete enumeration, random sampling)	**7, 8**
	10c		From whom consent was sought (representatives of the cluster, or individual cluster members, or both), and whether consent was sought before or after randomisation	**3; 7, 8; CONSORT flow diagram**
**Blinding**	11a	If done, who was blinded after assignment to interventions (for example, participants, care providers, those assessing outcomes) and how		**7, 15**
	11b	If relevant, description of the similarity of interventions		**15, see section 'control arm'**
**Statistical methods**	12a	Statistical methods used to compare groups for primary and secondary outcomes	How clustering was taken into account	**19, 20**
	12b	Methods for additional analyses, such as subgroup analyses and adjusted analyses		**19, 20**
**Results**				
**Participant flow (a diagram is strongly recommended)**	13a	For each group, the numbers of participants who were randomly assigned, received intended treatment, and were analysed for the primary outcome	For each group, the numbers of clusters that were randomly assigned, received intended treatment, and were analysed for the primary outcome	**Figure 1, CONSORT flow diagram**
	13b	For each group, losses and exclusions after randomisation, together with reasons	For each group, losses and exclusions for both clusters and individual cluster members	**7, 8 Figure 1**

**Recruitment**	14a	Dates defining the periods of recruitment and follow-up		**7, 8; Figure 2**
	14b	Why the trial ended or was stopped		**NA**
**Baseline data**	15	A table showing baseline demographic and clinical characteristics for each group	Baseline characteristics for the individual and cluster levels as applicable for each group	**NA (see 19, 20)**
**Numbers analysed**	16	For each group, number of participants (denominator) included in each analysis and whether the analysis was by original assigned groups	For each group, number of clusters included in each analysis	**NA (see 19, 20)**
**Outcomes and estimation**	17a	For each primary and secondary outcome, results for each group, and the estimated effect size and its precision (such as 95% confidence interval)	Results at the individual or cluster level as applicable and a coefficient of intracluster correlation (ICC or k) for each primary outcome	**NA (see 18)**
	17b	For binary outcomes, presentation of both absolute and relative effect sizes is recommended		-
**Ancillary analyses**	18	Results of any other analyses performed, including subgroup analyses and adjusted analyses, distinguishing pre-specified from exploratory		**NA (see 19, 20)**
**Harms**	19	All important harms or unintended effects in each group (for specific guidance see CONSORT for harms[3])		**NA (see 9)**
**Discussion**				
**Limitations**	20	Trial limitations, addressing sources of potential bias, imprecision, and, if relevant, multiplicity of analyses		**25, 26**
**Generalisability**	21	Generalisability (external validity, applicability) of the trial findings	Generalisability to clusters and/or individual participants (as relevant)	**25**

**Interpretation**	22	Interpretation consistent with results, balancing benefits and harms, and considering other relevant evidence	**23-26**
**Other information**			
**Registration**	23	Registration number and name of trial registry	**4**
**Protocol**	24	Where the full trial protocol can be accessed, if available	**NA**
**Funding**	25	Sources of funding and other support (such as supply of drugs), role of funders	**26, 27**

** Note: page numbers optional depending on journal requirements*

Table 2: Extension of CONSORT for abstracts[1,2] to reports of cluster randomised trials

Item	Standard Checklist item	Extension for cluster trials
**Title**	Identification of study as randomised	**Identification of study as cluster randomised**
**Trial design**	Description of the trial design (e.g. parallel, cluster, non-inferiority)	
**Methods**		
**Participants**	Eligibility criteria for participants and the settings where the data were collected	**Eligibility criteria for clusters**
**Interventions**	Interventions intended for each group	
**Objective**	Specific objective or hypothesis	**Whether objective or hypothesis pertains to the cluster level, the individual participant level or both**
**Outcome**	Clearly defined primary outcome for this report	**Whether the primary outcome pertains to the cluster level, the individual participant level or both**
**Randomization**	How participants were allocated to interventions	**How clusters were allocated to interventions**
**Blinding (masking)**	Whether or not participants, care givers, and those assessing the outcomes were blinded to group assignment	
**Results**		
**Numbers randomized**	Number of participants randomized to each group	**Number of clusters randomized to each group**
**Recruitment**	Trial status[1]	
**Numbers analysed**	Number of participants analysed in each group	**Number of clusters analysed in each group**
**Outcome**	For the primary outcome, a result for each group and the estimated effect size and its precision	**Results at the cluster or individual participant level as applicable for each primary outcome**
**Harms**	Important adverse events or side effects	
**Conclusions**	General interpretation of the results	
**Trial registration**	Registration number and name of trial register	
**Funding**	Source of funding	

[1] Relevant to Conference Abstracts

REFERENCES

[1] Hopewell S, Clarke M, Moher D, Wager E, Middleton P, Altman DG, et al. CONSORT for reporting randomised trials in journal and conference abstracts. *Lancet* 2008, 371:281-283

[2] Hopewell S, Clarke M, Moher D, Wager E, Middleton P, Altman DG at al (2008) CONSORT for reporting randomized controlled trials in journal and conference abstracts: explanation and elaboration. *PLoS Med* 5(1): e20

[3] Ioannidis JP, Evans SJ, Gotzsche PC, O'Neill RT, Altman DG, Schulz K, Moher D. Better reporting of harms in randomized trials: an extension of the CONSORT statement. *Ann Intern Med* 2004; 141(10):781-788.
