# Supplementary material for: The trial to reduce antimicrobial use in nursing home residents with Alzheimer’s disease and other dementias: study protocol for a cluster randomized controlled trial
Source: Trials. 2019 Oct 15;20:594. doi: 10.1186/s13063-019-3675-y (PMC6794759; doi:10.1186/s13063-019-3675-y)
Supplement: Supplementary file 3 — The Trial to reduce antimicrobial use in nursing home residents with Alzheimer’s disease and other dementias (TRAIN-AD) administrator survey. (PDF 87 kb) [file 13063_2019_3675_MOESM3_ESM.pdf]

Additional File 3. The Trial to Reduce Antimicrobial use In Nursing home residents with Alzheimer's disease and other Dementias (TRAIN-AD) Administrator Survey

Infection Management

1. Does your facility have an individual responsible for infection control?

☐ Yes
☐ No
☐ Do not know

1a. If yes, what are this person's responsibilities in addition to infection control? *Please mark all that apply.*

☐ Medical Director or Assistant Director
☐ Director/Assistant Director of Nursing Services
☐ Consultant Pharmacist
☐ Quality Coordinator/ Quality Management
☐ Staff Education/Staff Development
☐ Staff Nurse
☐ Employee Health
☐ Other *(please explain)*
☐ No other activities

2. Does your facility have an antibiotic stewardship program?

☐ Yes
☐ No
☐ Do not know

2a. If yes, indicate who is accountable for stewardship activities? *Please select all that apply.*

☐ Medical Director or Assistant Director
☐ Director/Assistant Director of Nursing Services
☐ Consultant Pharmacist
☐ Quality Coordinator/ Quality Management
☐ Staff Education/Staff Development
☐ Staff Nurse
☐ Employee Health
☐ Other *(please explain)*
☐ Do not know

3. Which of the following policies or programs are in place at your facility? *Please mark all that apply.*

   - ☐ Collect data on antibiotic use
   - ☐ Antibiotic prescribing guidelines or algorithms for lower respiratory tract infections (LRIs)
   - ☐ Antibiotic prescribing guidelines or algorithms for urinary tract infections (UTIs)
   - ☐ Restrict use of specific antibiotics
   - ☐ Review cases to assess appropriateness of antibiotic administration and/or indication
   - ☐ Provide feedback to clinicians on antibiotic use and prescribing
   - ☐ Provide education resources for improving antibiotic use
   - ☐ Other *(please explain)*
   - ☐ Do not know

4. Does your facility currently use any standardized protocols or initiatives (e.g., reference algorithms) for diagnosing and treatment of UTIs?

   - ☐ Yes
   - ☐ No
   - ☐ Refused to answer
   - ☐ Do not know

   4a. If yes, what specific protocols or initiatives for UTI management are used in your facility?

   - ☐ Interact CARE PATH for treatment of symptoms of UTIs
   - ☐ Massachusetts state initiative using the SBAR Protocol for Diagnosing UTIs in long-term care (LTC) environments
   - ☐ Mass Coalition’s ABCs for diagnosing UTIs in LTC
   - ☐ Other

5. Does your facility currently use any standardized protocols or initiatives (e.g., reference algorithms) for diagnosing and treatment of LRIs?

   - ☐ Yes
   - ☐ No
   - ☐ Refused to answer
   - ☐ Do not know

5a. If yes, what specific protocols or initiatives for LRI management are used in your facility?

- ☐ Interact CARE PATH for treatment of symptoms of LRIs
- ☐ Massachusetts state initiative using the SBAR Protocol for Diagnosing LRIs in long-term care (LTC) environments
- ☐ Mass Coalition's ABCs for diagnosing LRIs in LTC
- ☐ Other

Capabilities

6. Does your facility have the capability to do chest x-rays on site?

- ☐ Yes
- ☐ No
- ☐ Do not know

7. Does your facility have the capability to manage infections on-site by administrating intravenous antibiotics?

- ☐ Yes
- ☐ No
- ☐ Do not know

8. Please indicate how often your facility has **on-site** access to the following staff. *Please select <u>one</u> response in each row.*	**Full-time**	**Part-time**	**On call**	**No access**
a. A physician	☐	☐	☐	☐
b. A physician assistant	☐	☐	☐	☐
c. An advanced practice registered nurse, which includes nurse practitioners	☐	☐	☐	☐

End-of-Life Care Practices and Processes

	**Rarely**	**Occas-ionally**	**Often**	**Almost always**
9. How often do the LTC units in your facility use MOLST?	☐	☐	☐	☐
10. How often do the LTC units in your facility use INTERACT Advance Care Planning tools?	☐	☐	☐	☐

11. Do the LTC units in your facility routinely use any other standardized documents to document advance directives?

- ☐ No
- ☐ Yes *(please describe)*
- ☐ Do not know

	**Rarely**	**Occas-ionally**	**Often**	**Almost always**
12. How often does a resident with advanced dementia in your facility get referred to hospice?	☐	☐	☐	☐
13. How often does a resident with advanced dementia in your facility get a consultation from a palliative care specialist other than through hospice?	☐	☐	☐	☐

14. In your facility, how often are preferences for infection management discussed with proxies of residents with advanced dementia?	**Rarely**	**Some-times**	**Often**	**Almost always**
a. On admission	☐	☐	☐	☐
b. During regular care plan meetings	☐	☐	☐	☐
c. When a resident develops a fever or other sign/symptom of an infection	☐	☐	☐	☐
d. Following an event such as an aspiration	☐	☐	☐	☐
